# A Model to Detect Autochthonous Group 1 and 2 Brazilian *Vaccinia virus* Coinfections: Development of a qPCR Tool for Diagnosis and Pathogenesis Studies

**DOI:** 10.3390/v10010015

**Published:** 2017-12-30

**Authors:** Rafael Calixto, Graziele Oliveira, Maurício Lima, Ana Cláudia Andrade, Giliane de Souza Trindade, Danilo Bretas de Oliveira, Erna Geessien Kroon

**Affiliations:** 1 Laboratório de Vírus, Departamento de Microbiologia, Universidade Federal de Minas Gerais, Belo Horizonte 31270-901, Minas Gerais, Brazil; calixtomicro@yahoo.com.br (R.C.); graziufmg@yahoo.com.br (G.O.); maurili15@hotmail.com (M.L.); ana.andrade2008@hotmail.com (A.C.A.); gitrindade@yahoo.com.br (G.d.S.T.); danilobretas@yahoo.com.br (D.B.d.O.); 2 Faculdade de Medicina de Diamantina, Universidade Federal dos Vales do Jequitinhonha e Mucuri, Dimantina 39100-000, Minas Gerais, Brazil

**Keywords:** *Vaccinia virus*, qPCR, coinfection, mice model

## Abstract

*Vaccinia virus* (*VACV*) is the etiological agent of bovine vaccinia (BV), an emerging zoonosis that has been associated with economic losses and social effects. Despite increasing reports of BV outbreaks in Brazil, little is known about the biological interactions of Brazilian *VACV* (*VACV*-BR) isolates during coinfections; furthermore, there are no tools for the diagnosis of these coinfections. In this study, a tool to co-detect two variants of *VACV* was developed to provide new information regarding the pathogenesis, virulence profile, and viral spread during coinfection with *VACV*-BR isolates. To test the quantitative polymerase chain reactions (qPCR) tool, groups of BALB/c mice were intranasally monoinfected with Pelotas virus 1—Group II (PV1-GII) and Pelotas virus 2—Group I (PV2-GI), or were coinfected with PV1-GII and PV2-GI. Clinical signs of the mice were evaluated and the viral load in lung and spleen were detected using simultaneous polymerase chain reactions (PCR) targeting the *A56R* (*hemagglutinin*) gene of *VACV*. The results showed that qPCR for the quantification of viral load in coinfection was efficient and highly sensitive. Coinfected mice presented more severe disease and a higher frequency of *VACV* detection in lung and spleen, when compared to monoinfected groups. This study is the first description of PV1 and PV2 pathogenicity during coinfection in mice, and provides a new method to detect *VACV*-BR coinfections.

## 1. Introduction

*Vaccinia virus* (*VACV*) is a member of the family *Poxviridae*, genus *Orthopoxvirus*, which includes other members, such as *Variola virus*, *Cowpox virus* and *Monkeypox virus* [1,2]. *Variola virus* was one of the most terrible pathogens in human history, but it was declared eradicated in 1980 after an intensive vaccination campaign promoted by the World Health Organization (WHO) [3]. *VACV* can induce serological cross-reactivity against other orthopoxvirus (OPV) members and was used in the WHO campaign [1,3].

*VACV* is the etiological agent of bovine vaccinia (BV), an exanthematous disease that causes ulcerative lesions in cattle and humans, economic losses, and social effects in South America and Asia, especially in Brazil [4,5,6,7,8]. The clinical signs of BV range from papules and vesicles to scabs, mainly on the udder and teats of bovines. In humans, lesions occur primarily on the hands and arms, and other symptoms, such as fever, myalgia, headache, arthralgia, and lymphadenopathy, have been described and there is a significant economic impact on rural workers [1,6].

The natural circulation of *VACV* in Brazil has been often reported since 1999, and is associated with exanthematous outbreaks [6,7,9,10,11,12]. Many studies have shown biological and genetic variations among Brazilian *VACV* (*VACV*-BR) isolates. This variability allowed *VACV*-BR clustering into two distinct groups: Group 1 (GI) and group 2 (GII). These two groups are supported by biological features, such as virulence in a BALB/c mouse model and plaque phenotype in BSC-40 cells. GII isolates display larger plaque sizes and are virulent to mice, unlike GI [4,12,13,14,15,16]. Furthermore, molecular diversity is observed in specific *VACV* genes, such as the *hemagglutinin* gene (*A56R*), *A-type inclusion body* gene (*A26L*), and *chemokine-binding protein* gene (*C23L*), and these genes have been used in phylogenetic studies and further confirmed the dichotomy between GI and GII *VACV*-BR. The *A56R* sequence contains a signature deletion of 18 nt, present in the sequences of GI isolates and absent of GII isolates, which is used as a “molecular marker” for *VACV*-BR group identification [9,13,16,17,18]. The circulation of the two *VACV-BR* groups was demonstrated in the same outbreak in 2006 [4] and in the same host as a coinfection was only identified later [15,16]. In 2008, a *VACV* outbreak, caused by viruses of the two *VACV-BR* groups, was described in horses from Pelotas City, Brazil. This coinfection presented hemorrhagic lesion and scabs in the muzzles and nostrils of animals [15,19]. The *VACV* isolates were named Pelotas virus 1—Group II (PV1-GII) and Pelotas virus 2—Group I (PV2-GI) and were used in this study [15]. Despite increasing reports of outbreaks related to *VACV*-BR, little is known about its biological relevance, virulence profile and viral spread during coinfections with *VACV-BR* of GI and GII. Moreover, until now, there have been no established tools for the diagnosis or pathogenesis studies of coinfections with *VACV-BR* of GI and GII. As the best well-characterized molecular difference of the two groups is the *A56R* sequence which contains a signature deletion of 18 nt for GI, it results in the difficulty of developing a test for direct quantification of this group.

In this study, a new tool for the detection and quantification of *VACV* isolates in coinfections was developed and could be used as a tool to provided new information regarding the diagnosis, pathogenicity, virulence profile, and viral spread during a coinfection with *VACV*-BR isolates. Our method aims to improve screening in outbreaks and consequently the study of *VACV*-BR GI/GII coinfections pathogenesis.

## 2. Materials and Methods

### 2.1. Ethical Statement

This study was approved by the Committee of Ethics in Animal Use from the Universidade Federal de Minas Gerais (CEUA/UFMG, Belo Horizonte, Brazil), protocol number 207/2010.

### 2.2. Cells and Viruses

African green monkey kidney BSC-40 (ATCC-CRL-2761) and VERO (ATCC-CCL-81) cells were maintained in a 5% CO_2_ atmosphere at 37 °C, in Eagle’s Minimum Essential Medium (MEM) (Gibco BRL, Invitrogen, Carlsbad, CA, USA), supplemented with 5% fetal bovine serum (FBS) (Cultilab, Brazil), 25 µg/mL fungizone (Amphotericin B) (Cristália, São Paulo, Brazil), 500 U/mL penicillin and 50 µg/mL gentamicin (Schering-Plough, São Paulo, Brazil). VERO cells were used for viral replication and the BSC-40 cells were used for viral plaque phenotypes and titration. The *VACV* used in the study, PV1 and PV2, were isolated from clinical specimens of horses during an equine vaccinia outbreak [15,19].

### 2.3. Animal Experiments

For all animal experiments, five-week-old male Balb/c mice were used, and were maintained in micro-isolators located in a ventilated animal caging system (Alesco Ltd., Campinas, SP, Brazil), and were provided with commercial mouse food and water, ad libitum, in controlled lighting (12 h light–12 h dark), humidity (60–80%) and temperature (22 ± 1 °C). The mice of all groups were anesthetized via intraperitoneal injection of 0.1 mg of ketamine and 0.01 mg of xylazine in 0.9% PBS, per gram of animal weight. The four groups of five mice were inoculated intranasally with 10 µL of viral suspension: PV1-GII 1 × 10^6^ p.f.u.; PV2-GI 1 × 10^6^ p.f.u.; PV1-GII+ PV2-GI 5 × 10^5^ p.f.u. of each sample; a negative control group was inoculated with 10 μL of PBS, as previously described [14]. Mice were weighed daily, and other clinical signs were recorded for 30 days post infection (d.p.i.). To study viral tropism, the mice were euthanized with an overdose of anesthetics (three times the anesthetic solution) and were perfused with PBS-EDTA intracardiac and those animals had their spleens and lungs collected on 5 d.p.i. [14]. The collected organs were weighed and macerated with the Beadbeater 16 homogenizer in 500 µL of PBS, with glass beads in the microtubes thread. Three cycles of freezing and thawing were performed in order to release the viral particles from the cells. The cells were then centrifuged at 425× *g* for 10 min at 4 °C and the supernatants were used for DNA extraction and plaque phenotype assays. For DNA extraction, the High Pure Viral Nucleic Acid Kit (Roche Diagnostics GmbH, Branchburg, NJ, USA) was used.

### 2.4. Plaque Phenotype

For plaque phenotype assays, BSC40 cells seeded in 6-well plates at 90–95% confluence were inoculated with the macerated tissues. After 1 h of adsorption (37 °C, 5% CO_2_), monolayers were washed twice with PBS and overlaid with solid medium, prepared by mixing equal parts (1:2) of 1% agarose and twice the standard concentration Eagle’s minimum essential medium (MEM) supplemented with 2% FBS. After 48 h of incubation (37 °C, 5% CO_2_), cells were fixed with formaldehyde and stained with crystal violet for plaque size analysis.

### 2.5. qPCR

The qPCR consisted of two simultaneous reactions, A and B, targeting the *hemagglutinin* gene [20]. DNA amplifications were carried out in duplicate in a StepOne™ thermocycler (Applied Biosystems, Foster City, CA, USA). qPCR took place in a total volume of 10 µL, containing 5 µL of Master Mix SYBR Green (Applied Biosystems, manufactured by Roche, Branchburg, NJ, USA), 200 nM of each of the forward and reverse primers (Table 1) and 10–50 ng of each DNA sample.

**Figure 1:**
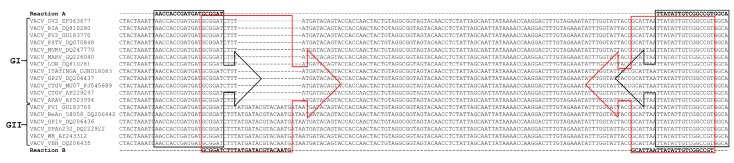
Alignment of the target DNA region within the *A56R* gene of the *Vaccinia virus* used for primer sequence design. The virus sequences were obtained from GenBank and the accession numbers are shown in the figure. The alignment was performed using the standard parameters of CLUSTAL W. The primers used in Reaction A (*A56R*-gen F and *A56R*-gen R) are outlined in black and, for Reaction B (*A56R*-BVV-nDEL F and *A56R*-generic R), are in red.

Figure 1 shows the target DNA regions for the primers used in this study. Thermal cycling conditions were as follows: one cycle at 95 °C for 10 min, followed by 40 cycles at 95 °C for 10 s, and 58 °C for 40 s; and a melting curve analysis, consisting of 95 °C for 15 s, 58 °C for 15 s, followed by increasing the temperature by 1 °C every 2 s until 95 °C was reached, and then 95 °C for 15 s. Standard curves were constructed by plotting four dilutions of each prototype DNA against the corresponding cycle threshold value (Ct); melting curves were used to ensure there were no non-specific amplifications.

For standardization, a curve was made using lungs from uninfected Balb/c mice, spiked with 10^6^, 10^5^, 10^4^, 10^3^, 10^2^ or 10^1^ p.f.u. of PV1-GII and PV2-GI. For validation lungs from uninfected Balb/c mice were spiked with 10^5^ p.f.u. of each sample (PV1-GII and PV2-GI). DNA was extracted and qPCR reactions A and B were performed. An equation for the differential viral load calculation was proposed:

[ ] *VACV* GI = [ ] *VACV* total (GI+GII) − [ ] *VACV* GII
[ ] *VACV* GI = 3.38 × 10^6^ − 1.68 × 10^6^
[ ] *VACV* GI = 1.70 × 10^6^
GI = 1.70 × 10^6^ and GII = 1.68 × 10^6^

### 2.6. Statistical Analyses

All results were plotted using GraphPad Prism (GraphPad Software, version 6.01, La Jolla, CA, USA) and compared using one-way ANOVA and Tukey’s multiple comparisons test, and two-way ANOVA using the Bonferroni method. In all tests, *p*-values < 0.05 were considered statistically significant.

## 3. Results

### 3.1. Development of qPCR Tool

**Figure 2:**
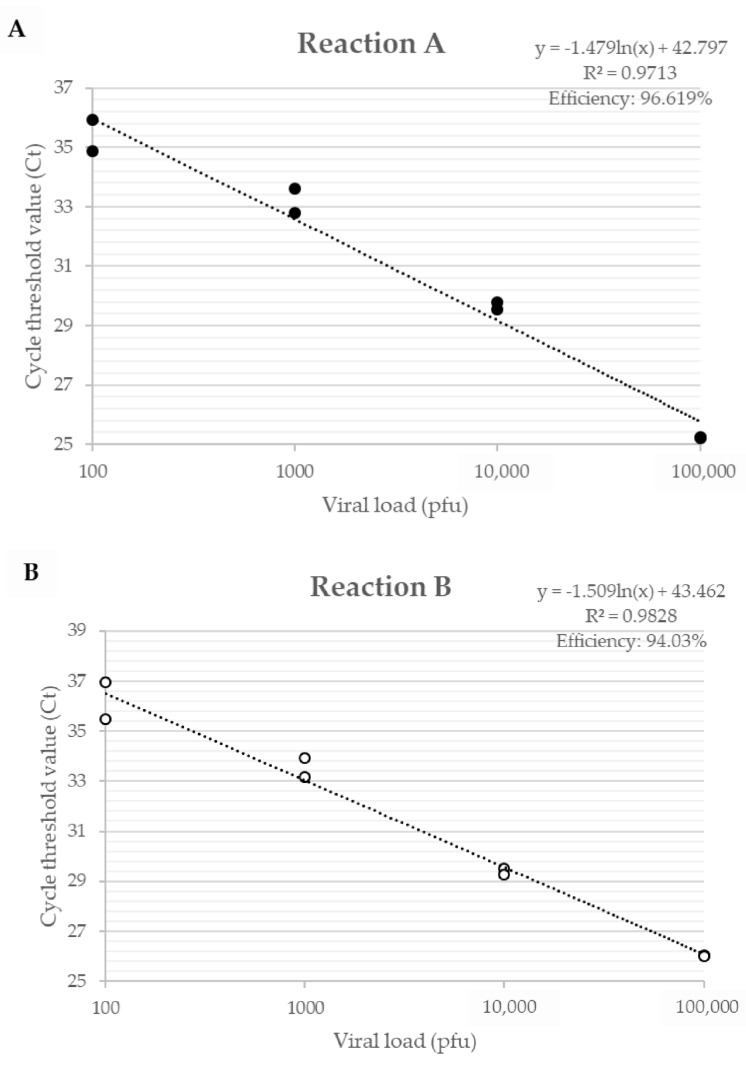
Efficiency curve of *A56R* gene qPCR in coinfection of PV1-GII and PV2-GI. Uninfected Balb/c mice lungs were spiked with 10^6^, 10^5^, 10^4^, 10^3^, 10^2^ and 10^1^ p.f.u. of PV1-GII and PV2-GI. (**A**) in **Reaction A shown in black circles**, primers *A56R*-gen F and *A56R*-gen R were used; and (**B**) in **Reaction B shown in white circles**, primers *A56R*-BVV-nDEL F and *A56R*-generic R were used.

The efficiency of primers developed for the *A56R* gene in *VACV* coinfection, for Reaction A, showed an efficiency of 96.6% and an R² value of 0.971; for Reaction B, an efficiency of 94.0% and a value of R² of 0.983 (Figure 2) were found. The detection limit of the assay was 2 p.f.u./mg of tissue and the reaction was efficient in different matrices such as lung, gut, and spleen.

The analysis of only one quantitative PCR (qPCR) peak fluorescence reaction was not able to distinguish the *VACV*-BR groups and to identify the presence of the deletion of 18 nt in *A56R* gene of GI viruses. In qPCR Reaction A, the fluorescence peaks were within 0.5 °C, making it difficult to rely on the melting (Tm) curve as a unique identifier for each PCR product (Tm GI: 75.8 °C and Tm GII: 76.3 °C). In Reaction B, GI is not amplified.

### 3.2. A56R qPCR as a Tool to Study Pathogenesis and Viral Spread

#### 3.2.1. Clinical Signs in Mice: Coinfected Versus Monoinfected

**Figure 3:**
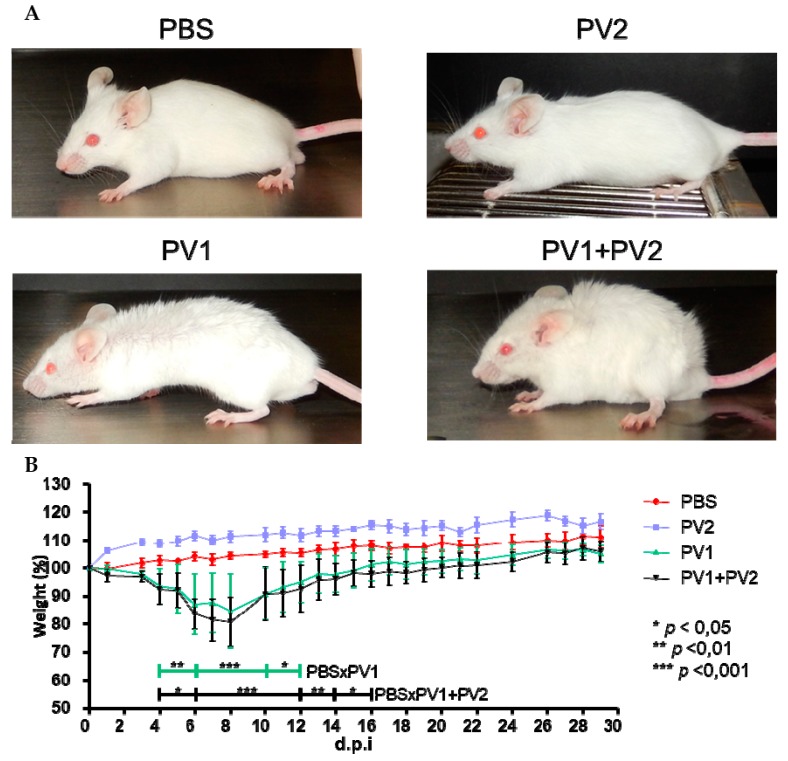
Clinical signs of Balb/c mice coinfected and monoinfected with PV1-GII and PV2-GI. Five-week-old male Balb/c mice were intranasally inoculated with 10 µL of viral suspension: PV1-GII 1 × 10^6^ p.f.u.; PV2-GI 1 × 10^6^ p.f.u.; PV1-GII + PV2-GI 5 × 10^5^ p.f.u. of each sample; and a negative control group was inoculated with 10 μL of PBS. Clinical signs were observed on day 5 p.i and were recorded for 30 days post infection (d.p.i.). (**A**) In PBS and PV2-GI groups, no clinical signals were observed. Mice monoinfected with PV1-GII and coinfected (PV1-GII + PV2-GI) showed fur ruffling and hunching of the back. (**B**) The mice were daily weighed and relative mean weight was calculated. The error bars indicate standard deviations. PV1-GII: Pelotas virus 1 Group II; PV2-GI: Pelotas virus 2 Group I; PBS: phosphate-buffered saline. Asterisks indicate a statistically significant difference: * *p* < 0.05; ** *p* < 0.01; *** *p* < 0.001.

Mice that were inoculated with PV1-GII (1 × 10^6^) and coinfected (PV1-GII + PV2-GI with 5 × 10^5^ of each) showed the first clinical signs, such as facial edema, fur ruffling, hunching of the back, dyspnea and severe weight loss from 4 to 15 d.p.i. The coinfected mice presented a slightly longer clinical manifestation compared to the PV1-GII group, which was not significant with respect to percent weight loss (PV1-GII vs. PV1-GII + PV2-GI). In the PBS and PV2-GI groups, no clinical signs or weight loss were observed. The only significant differences were the weight variation of PV1-GII and the co-infected group compared to the PBS group (Figure 3).

#### 3.2.2. Coinfected Mice Present Higher Frequency of *VACV* Detection in Lungs and Spleens than Monoinfected Groups

**Figure 4:**
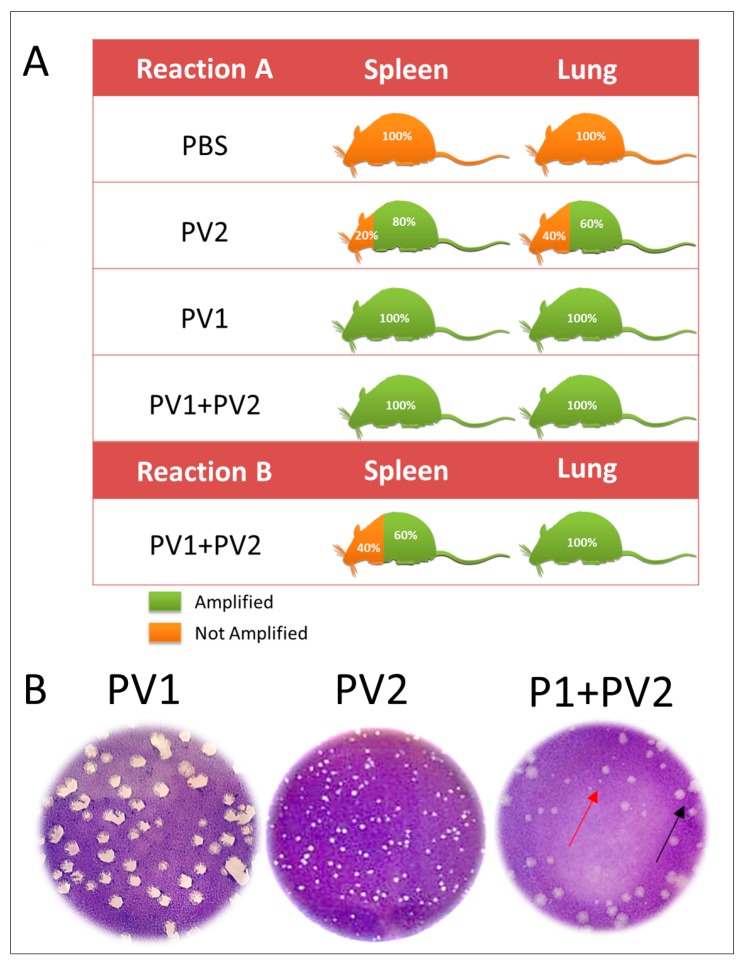
Detection of VAC-BR GI and GII in infected Balb/C mice: (**A**) qPCR detection of viral DNA from *VACV A56R* gene in mice spleen and lung samples. The results of Reaction A and Reaction B are represented. (**B**) Plaque phenotype assays of monoinfected (PV1-GII and PV2-GI) and coinfected mice showed two viral populations: small plaques-PV2 (red arrows) and large plaques-PV1 (black arrows). PV1-GII: Pelotas virus 1; PV2-GI: Pelotas virus 2; PBS: phosphate-buffered saline.

Using Reaction A, which amplifies all *VACV*, a positive qPCR was obtained in the lungs and spleen of 100 % of the mice inoculated with PV1- GII and PV1-GII + PV2-GI and in 60% of the PV2-GI infected mice lungs and 80% of the spleens. Reaction B of qPCR, which amplifies only the GII *A56R* gene (inoculated with PV1), showed a high positivity (100%) in lungs and only 60% of positivity in spleen (Figure 4A). This was confirmed by plaque phenotype assays (Figure 4B) of the lungs of four mice that were coinfected, which showed the presence of two viral populations (small plaques-PV2-GI and large plaques-PV1-GII).

#### 3.2.3. Viral Load in Lung and Spleen

**Figure 5:**
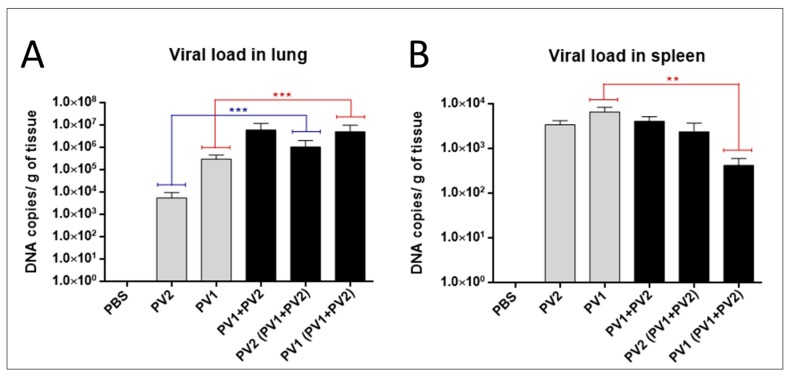
Viral load in mono or coinfected PV1-GII and PV2-GI mice. Viral loads in: lungs (**A**); and spleen (**B**) of Balb/c mice monoinfected (grey columns) and coinfected (black columns) with PV1-GII and PV2-GI were determined by qPCR. Comparisons between mono and coinfected groups are highlighted in red (PV1-GII groups) and blue (PV2-GI groups). The error bars indicate standard deviations. The statistical tests used were One-Way ANOVA and Tukey’s multiple comparisons test Asterisks indicate a statistically significant difference: ** *p* < 0.01; *** *p* < 0.001. PV1: Pelotas virus 1; PV2: Pelotas virus 2; PBS: phosphate-buffered saline.

The analysis of the viral loads in the lungs of mice in the monoinfected group (PV1) revealed an average viral load of almost two logs higher than monoinfected group (PV2-GI) (Figure 5A). A significant difference was observed when the viral loads of both monoinfected groups (PV1-GII and PV2-GI) were compared with each sample in coinfection (PV1-GII with PV1-GII + PV2-GI and PV2-GI with PV1-GII + PV2-GI) (Figure 5A). Differences about one log for PV1-GII groups and almost three logs between PV2-GI mice groups were observed (Figure 5A).

The differences between the monoinfected groups (PV1-GII or PV2-GI) in the spleens were irrelevant, demonstrating a higher concentration of viral DNA of GI in this tissue (Figure 5B). The viral load in spleens of PV2-GI monoinfected was very similar to that of PV2-GI from the coinfected group (PV1 + PV2) (Figure 5B). When the viral load of PV1-GII monoinfected with PV1-GI from the coinfected group (PV1-GII + PV2-GI) was compared, it showed a decrease of about one log with statistical significance (Figure 5B).

## 4. Discussion

Notifications of natural cases of *VACV* infection have increased [4,15,16], but this increase may be due to the development and improvement of study and detection techniques. qPCR is a rapid diagnostic tool, and is more sensitive than standard PCR; in addition, it allows acquiring molecular quantifications. Currently, multiplex real-time PCR has been described as a simple, reliable, and rapid method for the detection, identification and quantification of many kinds of viral coinfections [21,22,23]. In this way, qPCR assays have been used to detect and identify parapoxviruses and orthopoxviruses [24,25,26,27]. Here, we developed a new tool for the detection and quantification of *VACV*-BR isolates belonging to GI and GII during coinfections. This method was standardized under controlled conditions in animal model. Although the detection limit of 2 p.f.u./mg make its efficiency possible for many types of clinical samples more studies are needed to better clarify the total applications of method.

The qPCR assay to measure the viral load in coinfection was efficient and highly sensitive for different specimens (lung and spleen), showing a detection limit of 2 p.f.u./mg of tissue. The developed qPCR assay was useful as a detection system and in establishing the total viral load or relative viral load of *VACV*-GI and -GII in coinfections. The viral load could be measured because the efficiencies between two reactions are very close (96% and 94%).

Previous studies have shown that isolates of *VACV*-BR GII are virulent in murine model Balb/c, with weight loss and severe clinical signs, differently from GI, which is avirulent in this model [14]. Corroborating these data, the *VACV* isolates used in this work, in the monoinfected groups, PV1-GII and PV2-GI, followed the same pattern of virulence that has been previously described [15]. However, in this study, clinical signals in coinfected mice were evaluated and, unlike other studies conducted to date, this study evaluated animals until 30 d.p.i. when they had recovered from the infection [14,15,16]. Mice infected with the PV1-GII, a virulent representative of GII, and coinfected with PV2-GI, showed similar clinical signs, such as facial edema, fur ruffling, hunching of the back, dyspnea and severe weight loss. The weight variation data were significant at several points in the curve when we compared the control group to the groups infected with PV1-GII and co-infected (we use the two-way ANOVA test and Bonferroni method post-test).

The viral spread could be analyzed using this developed tool demonstrating a higher number of positive spleens samples (80%), compared to lung samples (60%) which is the primary site of inoculation in mice infected with PV2-GI. Furthermore, this analysis demonstrated the first description of spread of *VACV*-BR samples of GI. Viral loads data and the spread of *VACV*-BR GI isolates have not yet been demonstrated in other studies, and these results demonstrate the tendency of a higher tropism for lungs of PV1-GII and PV2-GI in coinfections.

In primary infection site (lungs), the coinfections with both viruses have higher viral loads, contrasting with monoinfected groups. On the other hand, in spleen the viral load of the virulent group (PV1-GII) is low in case of coinfection compared with the group monoinfected by same virus. These results may suggest an intriguing interaction among host and viruses which could help to understand why these two *VACV* groups are found in coinfections on nature. It is too early to answer the many questions raised by these results and more studies are needed to generalize these characteristics for all *VACV*-BR GI and GII coinfections, or as exclusive of PV1-GII and PV2-GI isolates.

Studies have shown that plaque size phenotypes are one of the biological characteristics that make possible the differentiation of the two groups of *VACV*-BR GI, with a small plaque phenotype, and GII with a large plaque phenotype [10,15,16,28]. Confirming biological characteristics of the plaque phenotype, the two viral populations (GI and GII) were detected in lungs of coinfected mice.

Using the developed tool of differential qPCR the first description of *VACV*-BR GI spread was possible. In previous studies, the GI virus could not be detected in mice lungs 5 d.p.i. [14]. Reports related to *VACV*-BR groups coinfections increases as well as the difficulty to detect both groups at the same time by methods of viral isolation and DNA sequencing. The main focus of this study was to develop an efficient tool to facilitate screening during outbreaks and consequently the study of coinfection, and also clarify its possible medical and veterinary importance.

## Figures and Tables

**Table 1:** Real-time PCR primers.

Reaction	Primers	Sequence (5’-3’)	Specificity
A	A56R-gen F	AACCACCGATGATGCGGAT	Amplify all VACV Group I and II.
	A56R-gen R	TGCCACGGCCGACAATATAA	
B	A56R-BVV-nDEL F	GCGGATCTTTATGATACGTACAATG	Amplify all VACV that do not present the 18nt deletion Group II [20].
	A56R-generic R	ACGGCCGACAATATAATTAATGC	
